# Supplementary material for: Social norms vs socioeconomic vulnerability: Gender identity and female labor force participation in Ecuador
Source: PLoS One. 2026 May 19;21(5):e0339503. doi: 10.1371/journal.pone.0339503 (PMC13186354; doi:10.1371/journal.pone.0339503)
Supplement: S1 Appendix — (DOCX) [file pone.0339503.s001.docx]

# **Appendix A**

**A1 Discontinuity tests**

*Figure A 1: Kernel density plot showing the distribution on either side of the cut-off point at 0.501, with a bin width of 5%.*


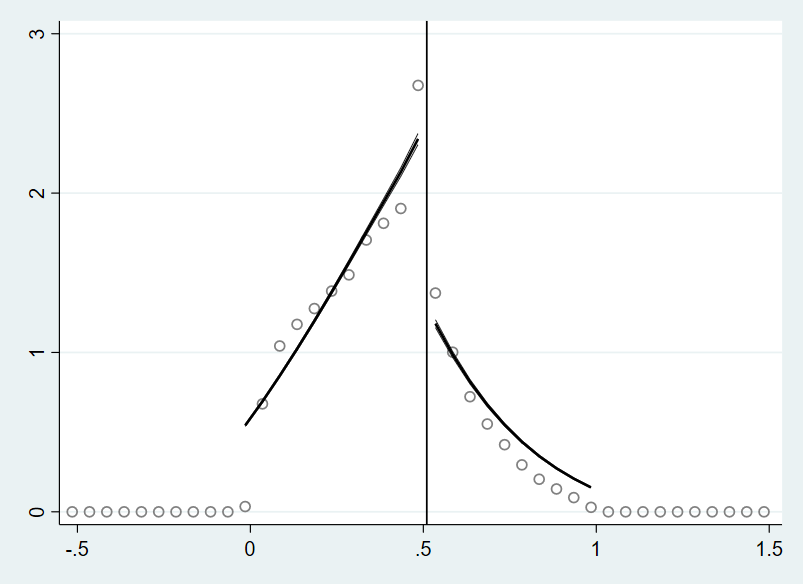


McCrary's (2008) density test was used to detect discontinuities at a cut-off of 0.501. The estimated discontinuity (log difference in height) is -0.664, with a p-value of 0.015, which indicates rejection of the null hypothesis of no discontinuity.

Figure A 2: Kernel density plot showing the 2007 distribution on either side of the cut-off point at 0.501, with a bin width of 5%

*
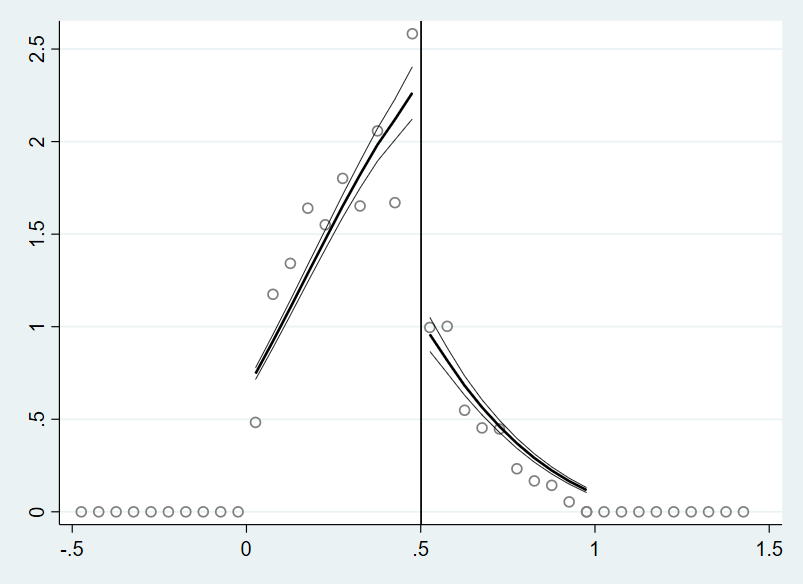
*

McCrary's (2008) density test was used to detect discontinuities at a cut-off of 0.501. The estimated discontinuity (log difference in height) is -0.8116, with a p-value of 0.063, which indicates rejection of the null hypothesis of no discontinuity.

Figure A 3: Kernel density plot showing the 2012 distribution on either side of the cut-off point at 0.501, with a bin width of 5%

*
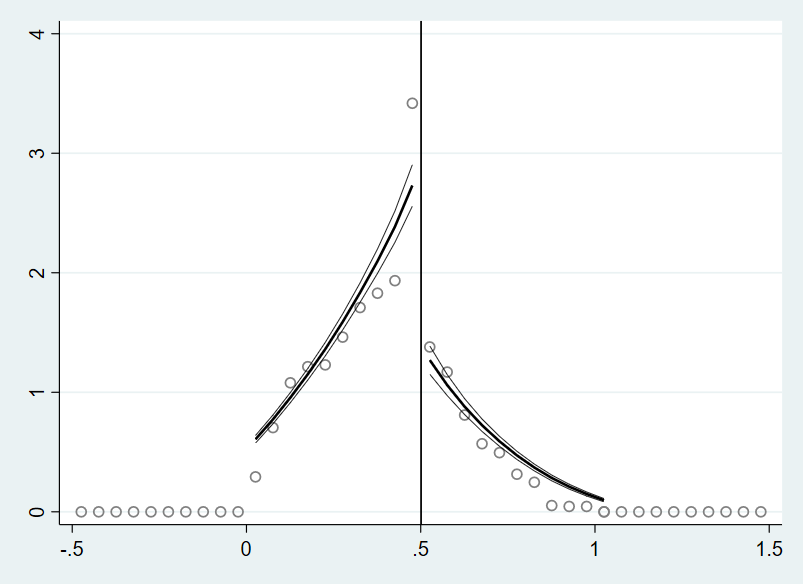
*

McCrary's (2008) density test was used to detect discontinuities at a cut-off of 0.501. The estimated discontinuity (log difference in height) is -0.744, with a p-value of 0.062, which indicates rejection of the null hypothesis of no discontinuity.

Figure A 4: Kernel density plot showing the 2017 distribution on either side of the cut-off point at 0.501, with a bin width of 5%

*
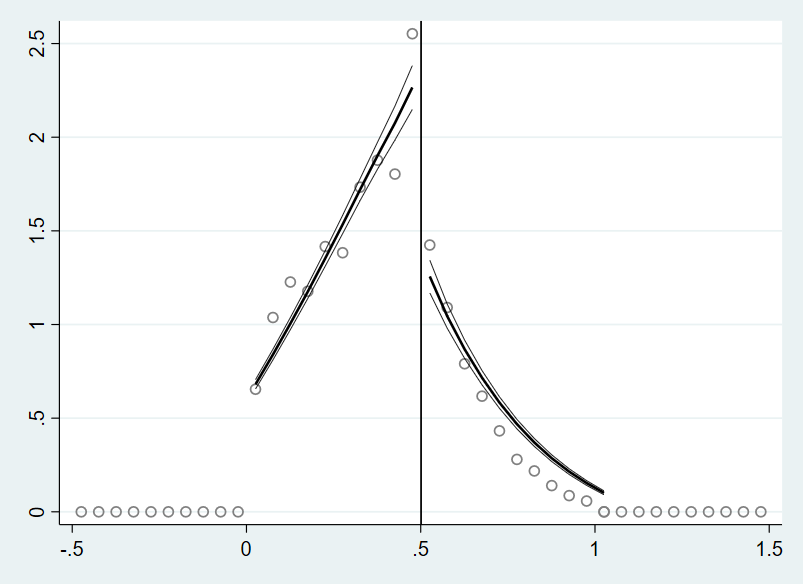
*

McCrary's (2008) density test was used to detect discontinuities at a cut-off of 0.501. The estimated discontinuity (log difference in height) is -0.551, with a p-value of 0.047, which indicates rejection of the null hypothesis of no discontinuity.

Figure A 5: Kernel density plot showing the 2022 distribution on either side of the cut-off point at 0.501, with a bin width of 5%

*
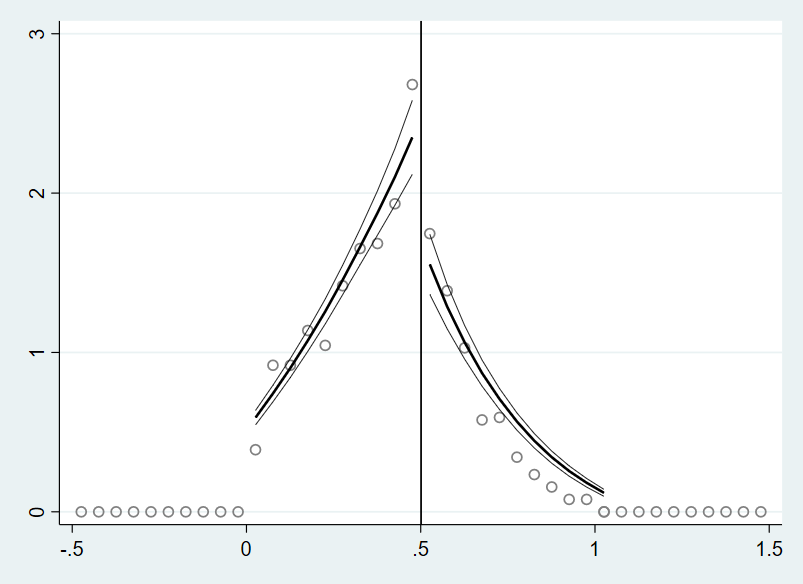
*

McCrary's (2008) density test was used to detect discontinuities at a cut-off of 0.501. The estimated discontinuity (log difference in height) is -0.384, with a p-value of 0.086, which indicates rejection of the null hypothesis of no discontinuity.

*Figure A 6: Histogram of the relative income distribution from 2007 to 2022, with a bin width of 1%.*


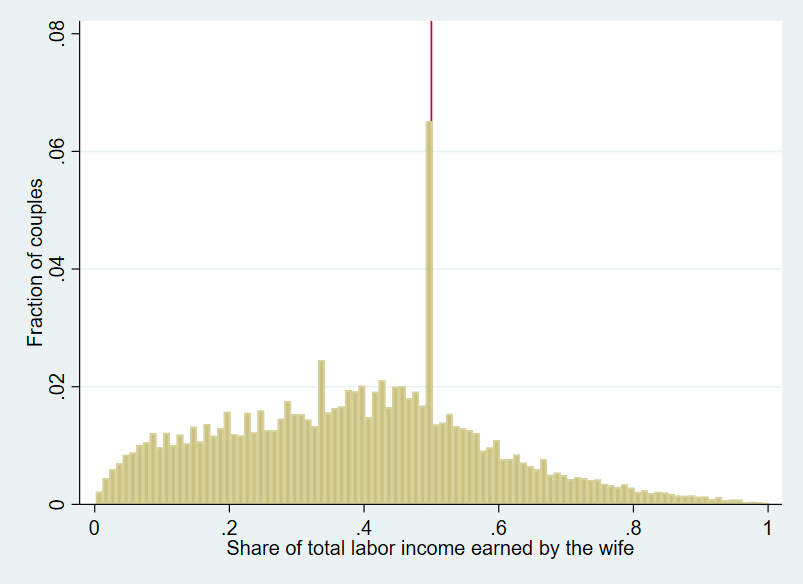


*Figure A 7: Kernel density plot showing the distribution on either side of the cut-off point at 0.501, with a bin width of 1%.*


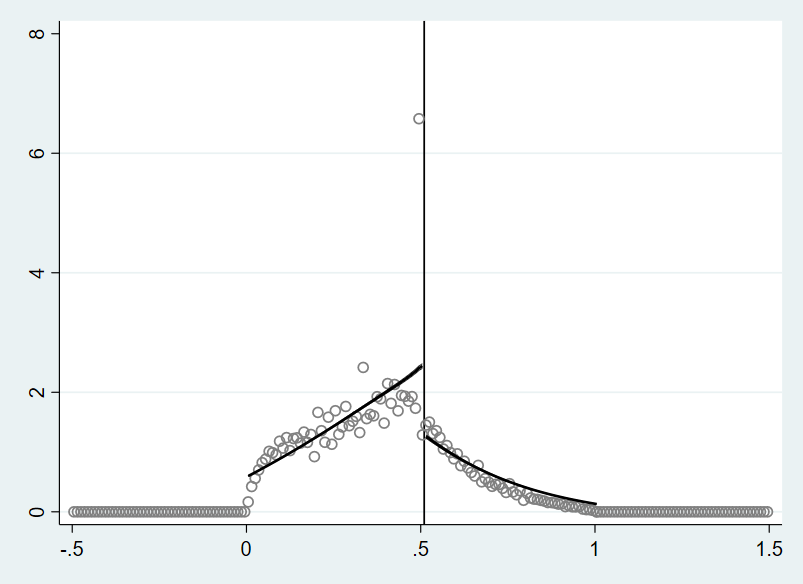


McCrary's (2008) density test was used to detect discontinuities at a cut-off of 0.501. The estimated discontinuity (log difference in height) is -0.67, with a p-value of 0.015, which indicates rejection of the null hypothesis of no discontinuity.

**A2 Alternative Part-time specification**

Table A 1: Potential relative income and different part-time job specifications

| Dependent variable Part-time Job (<=20 hours per week) | | | | | | | |
| --- | --- | --- | --- | --- | --- | --- | --- |
|  | (1) | | (2) | | (3) | | |
|  | Part-time | | Part-time | | Part-time | | |
| Prob. wife earns more | 0.041^***^ | | 0.037^***^ | | 0.037^***^ | | |
|  | (0.0056) | | (0.0065) | | (0.0065) | | |
| Observations | 69680 | | 69680 | | 69680 | | |
| *R*^2^ | 0.050 | | 0.050 | | 0.051 | | |
| Dependent variable Part-time Job (<=25 hours per week) | | | | | | | |
|  | (1) | | (2) | | (3) | | |
|  | Part-time | | Part-time | | Part-time | | |
| Prob. wife earns more | 0.051^***^ | | 0.046^***^ | | 0.046^***^ | | |
|  | (0.0060) | | (0.0071) | | (0.0071) | | |
| Observations | 69680 | | 69680 | | 69680 | | |
| *R*^2^ | 0.063 | | 0.063 | | 0.065 | | |
| Dependent variable Part-time Job (<=30 hours per week) | | | | | | | |
|  | (1) | | (2) | | (3) | | |
|  | Part-time | | Part-time | | Part-time | | |
| Prob. wife earns more | 0.062^***^ | | 0.056^***^ | | 0.056^***^ | | |
|  | (0.0067) | | (0.0081) | | (0.0081) | | |
| Observations | 69680 | | 69680 | | 69680 | | |
| *R*^2^ | 0.075 | | 0.075 | | 0.077 | | |
| Dependent variable Part-time Job (<=39 hours per week) | | | | | | | |
|  | | | (1) | | (2) | | (3) |
|  | | | Part-time | | Part-time | | Part-time |
| Prob. wife earns more | | | 0.067^***^ | | 0.061^***^ | | 0.061^***^ |
|  | | | (0.0067) | | (0.0081) | | (0.0081) |
| Observations | | | 69680 | | 69680 | | 69680 |
| *R*^2^ | | | 0.087 | | 0.087 | | 0.089 |

Source: Authors' own based work based on ENEMDU.

Robust standard errors in parenthesis are clustered at demographic group level.

+ p<0.10, * p<0.05, ** p<0.01, *** p<0.001

**A3 Robust checks estimations**

**A3.1 Alternative specification**

Table A 2: Potential relative income and job condition. Alternative specification.

|  | (1) | (2) | (3) | (4) |
| --- | --- | --- | --- | --- |
|  | Labor participation | Full-employment | Part-time | Tenure |
| Prob. wife earns more | 0.090^***^ | -0.12^***^ | 0.043^***^ | -0.11^***^ |
|  | (0.0064) | (0.0074) | (0.0073) | (0.0065) |
| Observations | 131607 | 71693 | 71693 | 71693 |
| *R*^2^ | 0.115 | 0.266 | 0.085 | 0.207 |

Prob. wife earns more variable constructed using non-married/ non-cohabit womens income

Source: Authors' own work based on ENEMDU.

Robust standard errors in parenthesis are clustered at demographic group level.

+ p<0.10, * p<0.05, ** p<0.01, *** p<0.001

Table A 3: Potential relative income and jobs condition by education level. Alternative specification.

|  | (1) | (2) | (4) | (5) |
| --- | --- | --- | --- | --- |
|  | Labor participation | Formal | Part-time | Tenure |
| Prob. wife earns more | 0.10^***^ | -0.046^*^ | 0.038^**^ | -0.13^***^ |
|  | (0.0091) | (0.02) | (0.013) | (0.0093) |
| Observations | 72903 | 36012 | 36012 | 36012 |
| *R*^2^ | 0.116 | 0.076 | 0.035 | 0.034 |
|  | (1) | (2) | (4) | (5) |
|  | Labor participation | Formal | Part-time | Tenure |
| Prob. wife earns more | 0.10^***^ | -0.12^***^ | 0.081^***^ | -0.10^***^ |
|  | (0.0091) | (0.017) | (0.016) | (0.016) |
| Observations | 72903 | 16244 | 16244 | 16244 |
| *R*^2^ | 0.116 | 0.089 | 0.043 | 0.034 |
|  | (1) | (2) | (4) | (5) |
|  | Labor participation | Formal | Part-time | Tenure |
| Prob. wife earns more | 0.000042 | -0.059^***^ | 0.064^***^ | -0.12^***^ |
|  | (0.013) | (0.011) | (0.013) | (0.018) |
| Observations | 23554 | 17414 | 17414 | 17414 |
| *R*^2^ | 0.033 | 0.039 | 0.023 | 0.070 |

Prob. wife earns more variable constructed using non-married/ non-cohabit women’s income

Source: Authors' own work based on ENEMDU.

Robust standard errors in parenthesis are clustered at demographic group level.

+ p<0.10, * p<0.05, ** p<0.01, *** p<0.001

**A3.2 By children in the household**

Table A 4: Potential relative income and job condition by households with and without children

|  | (1) | (2) | (3) | (4) |
| --- | --- | --- | --- | --- |
|  | Labor participation | Formal | Part-time | Tenure |
| Sample restricted to households with children <= 12 y.o | | | | |
| Prob. wife earns more | 0.092^***^ | -0.079^***^ | 0.042^***^ | -0.11^***^ |
|  | (0.0094) | (0.0097) | (0.012) | (0.0098) |
| Observations | 74222 | 39251 | 39251 | 39251 |
| *R*^2^ | 0.117 | 0.338 | 0.081 | 0.194 |
| Sample restricted to households without children <= 12 y.o | | | | |
| Prob. wife earns more | 0.067^***^ | -0.10^***^ | 0.076^***^ | -0.11^***^ |
|  | (0.0097) | (0.010) | (0.011) | (0.0100) |
| Observations | 54787 | 30429 | 30429 | 30429 |
| *R*^2^ | 0.107 | 0.348 | 0.093 | 0.221 |

Source: Authors' own work based on ENEMDU.

Robust standard errors in parenthesis are clustered at demographic group level.

+ p<0.10, * p<0.05, ** p<0.01, *** p<0.001

Table A 5: Potential relative income and job condition by education level. Sample restricted to households with children <= 12 y.o

|  | (1) | (2) | (3) | (4) |
| --- | --- | --- | --- | --- |
|  | Labor participation | Formal | Part-time | Tenure |
| Primary | | | | |
| Prob. wife earns more | 0.12^***^ | -0.015 | -0.0010 | -0.12^***^ |
|  | (0.013) | (0.017) | (0.020) | (0.012) |
| Observations | 44254 | 21600 | 21600 | 21600 |
| *R*^2^ | 0.124 | 0.077 | 0.037 | 0.032 |
| Secondary | | | | |
| Prob. wife earns more | 0.053^*^ | -0.080^**^ | 0.046 | -0.15^***^ |
|  | (0.021) | (0.029) | (0.030) | (0.025) |
| Observations | 18075 | 8938 | 8938 | 8938 |
| *R*^2^ | 0.049 | 0.093 | 0.038 | 0.035 |
| University | | | | |
| Prob. wife earns more | 0.0074 | -0.048^**^ | 0.049^*^ | -0.075^*^ |
|  | (0.020) | (0.017) | (0.024) | (0.030) |
| Observations | 11893 | 8713 | 8713 | 8713 |
| *R*^2^ | 0.029 | 0.040 | 0.022 | 0.063 |

Source: Authors' own work based on ENEMDU.

Robust standard errors in parenthesis are clustered at demographic group level.

+ p<0.10, * p<0.05, ** p<0.01, *** p<0.001

Table A 6: Potential relative income and job condition by education level. Sample restricted to households without children <= 12 y.o

|  | (1) | | (2) | | (3) | (4) | |
| --- | --- | --- | --- | --- | --- | --- | --- |
|  | Labor participation | | Formal | | Part-time | Tenure | |
| Primary | | | | | | | |
| Prob. wife earns more | 0.12^***^ | | -0.034^+^ | | 0.036^+^ | -0.12^***^ | |
|  | (0.015) | | (0.018) | | (0.019) | (0.013) | |
| Observations | 28649 | | 14412 | | 14412 | 14412 | |
| *R*^2^ | 0.108 | | 0.077 | | 0.039 | 0.036 | |
| Secondary | | | | | | | |
| Prob. wife earns more | | 0.070^***^ | | -0.13^***^ | 0.100^***^ | | -0.15^***^ |
|  | | (0.018) | | (0.024) | (0.022) | | (0.025) |
| Observations | | 14470 | | 7308 | 7308 | | 8938 |
| *R*^2^ | | 0.049 | | 0.092 | 0.050 | | 0.035 |
| University | | | | | | | |
| Prob. wife earns more | | -0.024 | | -0.071^***^ | 0.085^***^ | | -0.16^***^ |
|  | | (0.020) | | (0.015) | (0.017) | | (0.025) |
| Observations | | 11668 | | 8709 | 8709 | | 8709 |
| *R*^2^ | | 0.044 | | 0.047 | 0.034 | | 0.086 |

Source: Authors' own work based on ENEMDU.

Robust standard errors in parenthesis are clustered at demographic group level.

+ p<0.10, * p<0.05, ** p<0.01, *** p<0.001

**A3.3 By sample period**

Table A 7: Potential relative income and job condition by time periods

|  | (1) | (2) | (3) | (4) |
| --- | --- | --- | --- | --- |
|  | Labor participation | Formal | Part-time | Tenure |
| Sample restricted to period 2007-2015 | | | | |
| Prob. wife earns more | 0.068^***^ | -0.094^***^ | 0.060^***^ | -0.11^***^ |
|  | (0.0081) | (0.0078) | (0.0097) | (0.0089) |
| Observations | 84667 | 43050 | 43050 | 43050 |
| *R*^2^ | 0.113 | 0.359 | 0.070 | 0.226 |
| Sample restricted to period 2016-2022 | | | | |
| Prob. wife earns more | 0.11^***^ | -0.11^***^ | 0.066^***^ | -0.099^***^ |
|  | (0.012) | (0.014) | (0.015) | (0.012) |
| Observations | 44342 | 26630 | 26630 | 26630 |
| *R*^2^ | 0.098 | 0.328 | 0.117 | 0.192 |

Source: Authors' own work based on ENEMDU.

Robust standard errors in parenthesis are clustered at demographic group level.

+ p<0.10, * p<0.05, ** p<0.01, *** p<0.001

Table A 8: Potential relative income and job condition by education level. Sample restricted to period 2007-2015

|  | (1) | (2) | (3) | (4) |
| --- | --- | --- | --- | --- |
|  | Labor participation | Formal | Part-time | Tenure |
| Primary | | | | |
| Prob. wife earns more | 0.11^***^ | -0.017 | 0.025 | -0.11^***^ |
|  | (0.011) | (0.016) | (0.018) | (0.011) |
| Observations | 51334 | 23632 | 23632 | 23632 |
| *R*^2^ | 0.107 | 0.081 | 0.024 | 0.031 |
| Secondary | | | | |
| Prob. wife earns more | 0.065^***^ | -0.10^***^ | 0.054^**^ | -0.097^***^ |
|  | (0.015) | (0.020) | (0.020) | (0.022) |
| Observations | 19298 | 9216 | 9216 | 9216 |
| *R*^2^ | 0.045 | 0.084 | 0.034 | 0.037 |
| University | | | | |
| Prob. wife earns more | -0.022 | -0.059^***^ | 0.060^***^ | -0.16^***^ |
|  | (0.017) | (0.014) | (0.015) | (0.023) |
| Observations | 14035 | 10202 | 10202 | 10202 |
| *R*^2^ | 0.037 | 0.040 | 0.033 | 0.058 |

Source: Authors' own work based on ENEMDU.

Robust standard errors in parenthesis are clustered at demographic group level.

+ p<0.10, * p<0.05, ** p<0.01, *** p<0.001

Table A 9: Potential relative income and job condition by education level. Sample restricted to period 2016-2022

|  | (1) | (2) | (3) | (4) |
| --- | --- | --- | --- | --- |
|  | Labor participation | Formal | Part-time | Tenure |
| Primary | | | | |
| Prob. wife earns more | 0.17^***^ | -0.020 | -0.011 | -0.14^***^ |
|  | (0.019) | (0.024) | (0.024) | (0.020) |
| Observations | 21569 | 12380 | 12380 | 12380 |
| *R*^2^ | 0.114 | 0.072 | 0.046 | 0.039 |
| Secondary | | | | |
| Prob. wife earns more | 0.091^**^ | -0.14^***^ | 0.084^*^ | -0.16^***^ |
|  | (0.028) | (0.035) | (0.033) | (0.029) |
| Observations | 13247 | 7030 | 7030 | 7030 |
| *R*^2^ | 0.043 | 0.109 | 0.047 | 0.041 |
| University | | | | |
| Prob. wife earns more | 0.018 | -0.064^***^ | 0.071^**^ | -0.058^*^ |
|  | (0.020) | (0.017) | (0.026) | (0.029) |
| Observations | 9526 | 7220 | 7220 | 7220 |
| *R*^2^ | 0.028 | 0.049 | 0.019 | 0.099 |

Source: Authors' own work based on ENEMDU.

Robust standard errors in parenthesis are clustered at demographic group level.

+ p<0.10, * p<0.05, ** p<0.01, *** p<0.001

**A4. Complete Set of Regression Coefficients**

*Table A 10: Complete Set of Coefficients for the Full Specifications Reported in Tables 3 and 4*

|  | (1) | (2) | (3) | (4) |
| --- | --- | --- | --- | --- |
|  | Labor participation | Formal | Part-time | Tenure |
| Prob. wife earns more | 0.079^***^ | -0.10^***^ | 0.060^***^ | -0.11^***^ |
|  | (0.0069) | (0.0070) | (0.0081) | (0.0072) |
| age 30-39 | 0.10^***^ | 0.011^+^ | -0.035^***^ | 0.015^*^ |
|  | (0.0078) | (0.0067) | (0.0074) | (0.0073) |
| age 40-49 | 0.13^***^ | -0.0090 | -0.034^***^ | 0.017^*^ |
|  | (0.0085) | (0.0080) | (0.0088) | (0.0083) |
| age 50-59 | 0.11^***^ | -0.030^**^ | -0.016 | 0.017^+^ |
|  | (0.0096) | (0.0093) | (0.010) | (0.0099) |
| High school | 0.044^***^ | 0.19^***^ | -0.049^***^ | 0.035^***^ |
|  | (0.0083) | (0.0081) | (0.0079) | (0.0074) |
| University | 0.35^***^ | 0.33^***^ | -0.11^***^ | 0.13^***^ |
|  | (0.024) | (0.019) | (0.020) | (0.022) |
| Minority | 0.040^***^ | -0.063^***^ | -0.015 | -0.0047 |
|  | (0.011) | (0.011) | (0.012) | (0.010) |
| highland | 0.22^***^ | 0.040^***^ | -0.087^***^ | 0.0093^*^ |
|  | (0.0056) | (0.0044) | (0.0052) | (0.0042) |
| Husband's income | 0.000010^*^ | 0.000058^***^ | -0.000010^*^ | -0.000019^***^ |
|  | (0.0000044) | (0.0000056) | (0.0000046) | (0.0000047) |
| hus. age 30-39 | 0.016^**^ | -0.012^+^ | 0.019^*^ | -0.015^*^ |
|  | (0.0052) | (0.0064) | (0.0079) | (0.0069) |
| hus. age 40-49 | 0.0029 | -0.017^*^ | 0.032^***^ | -0.030^***^ |
|  | (0.0060) | (0.0074) | (0.0086) | (0.0083) |
| hus. age 50-59 | -0.023^***^ | -0.013 | 0.025^**^ | -0.038^***^ |
|  | (0.0067) | (0.0083) | (0.0095) | (0.0090) |
| hus. edu. high school | 0.015^***^ | 0.100^***^ | -0.049^***^ | 0.042^***^ |
|  | (0.0042) | (0.0054) | (0.0052) | (0.0039) |
| hus. edu. university | 0.027^***^ | 0.13^***^ | -0.038^***^ | 0.055^***^ |
|  | (0.0054) | (0.0071) | (0.0062) | (0.0061) |
| hus. minority | 0.036^***^ | -0.038^***^ | -0.013 | -0.012 |
|  | (0.0097) | (0.011) | (0.012) | (0.0099) |
| Husband's income^2 | -3.7e-10 | -2.6e-09^***^ | 3.9e-10 | 7.8e-10^*^ |
|  | (2.6e-10) | (3.8e-10) | (2.4e-10) | (3.1e-10) |
| Husband's income^3 | 4.0e-15 | 2.6e-14^***^ | -4.0e-15 | -8.0e-15^*^ |
|  | (3.0e-15) | (4.7e-15) | (2.7e-15) | (3.8e-15) |
| Observations | 129009 | 69680 | 69680 | 69680 |
| *R*^2^ | 0.113 | 0.346 | 0.088 | 0.210 |

All regression include wifes' potential income at each 5th percentile and year dummies.

Source: Authors' own based work based on ENEMDU.

Robust standard errors in parenthesis are clustered at demographic group level.

+ p<0.10, * p<0.05, ** p<0.01, *** p<0.001

*Table A 11: Complete Set of Coefficients for the Specifications Reported in Table 5 (a)*

|  | (1) | (2) | (3) |
| --- | --- | --- | --- |
| Dependent variable  Labor participation | Primary | Secondary | University |
| Prob. wife earns more | 0.12^***^ | 0.074^***^ | -0.0060 |
|  | (0.0097) | (0.013) | (0.013) |
| age 30-39 | 0.098^***^ | 0.085^***^ | 0.082^***^ |
|  | (0.0086) | (0.010) | (0.011) |
| age 40-49 | 0.12^***^ | 0.11^***^ | 0.10^***^ |
|  | (0.0099) | (0.014) | (0.015) |
| age 50-59 | 0.10^***^ | 0.074^***^ | 0.059^**^ |
|  | (0.011) | (0.020) | (0.020) |
| Minority | 0.044^**^ | 0.012 | 0.054^**^ |
|  | (0.015) | (0.018) | (0.020) |
| highland | 0.28^***^ | 0.16^***^ | 0.060^***^ |
|  | (0.0070) | (0.0086) | (0.0070) |
| Husband's income | 0.000055^***^ | 0.000015^+^ | -0.000032^***^ |
|  | (0.0000092) | (0.0000082) | (0.0000065) |
| hus. age 30-39 | 0.016^*^ | -0.00022 | 0.044^***^ |
|  | (0.0077) | (0.0087) | (0.011) |
| hus. age 40-49 | 0.0040 | -0.0082 | 0.022^+^ |
|  | (0.0085) | (0.012) | (0.013) |
| hus. age 50-59 | -0.028^**^ | -0.023 | 0.012 |
|  | (0.0090) | (0.015) | (0.014) |
| hus. edu. high school | 0.013^*^ | 0.014^*^ | 0.011 |
|  | (0.0063) | (0.0066) | (0.0096) |
| hus. edu. university | 0.029^**^ | 0.012 | 0.039^***^ |
|  | (0.011) | (0.0093) | (0.0095) |
| hus. minority | 0.040^**^ | 0.036^*^ | -0.029 |
|  | (0.013) | (0.017) | (0.019) |
| Husband's income^2 | -4.0e-09^***^ | -1.8e-10 | 1.3e-09^***^ |
|  | (9.8e-10) | (7.4e-10) | (3.0e-10) |
| Husband's income^3 | 5.6e-14^**^ | 5.8e-16 | -1.1e-14^***^ |
|  | (1.7e-14) | (9.3e-15) | (3.1e-15) |
| Observations | 72903 | 32545 | 23561 |
| *R*^2^ | 0.117 | 0.046 | 0.033 |

All regression include wifes' potential income at each 5th percentile and year dummies.

Source: Authors' own based work based on ENEMDU.

Robust standard errors in parenthesis are clustered at demographic group level.

+ p<0.10, * p<0.05, ** p<0.01, *** p<0.001

*Table A 12: Complete Set of Coefficients for the Specifications Reported in Table 5 (b)*

|  | (1) | (2) | (3) |
| --- | --- | --- | --- |
| Dependent variable Formal Sector | Primary | Secondary | University |
| Prob. wife earns more | -0.031^*^ | -0.13^***^ | -0.061^***^ |
|  | (0.013) | (0.017) | (0.011) |
| age 30-39 | 0.0050 | 0.016 | 0.0028 |
|  | (0.0074) | (0.013) | (0.0100) |
| age 40-49 | -0.025^**^ | 0.0030 | -0.0044 |
|  | (0.0090) | (0.017) | (0.013) |
| age 50-59 | -0.027^*^ | -0.023 | -0.022 |
|  | (0.011) | (0.023) | (0.018) |
| Minority | -0.036^*^ | -0.037 | -0.029 |
|  | (0.014) | (0.026) | (0.019) |
| highland | 0.0098^*^ | 0.092^***^ | 0.033^***^ |
|  | (0.0048) | (0.0096) | (0.0063) |
| Husband's income | 0.00025^***^ | 0.000085^***^ | 0.000012^***^ |
|  | (0.000019) | (0.0000099) | (0.0000032) |
| hus. age 30-39 | -0.031^***^ | -0.017 | 0.020^+^ |
|  | (0.0091) | (0.013) | (0.011) |
| hus. age 40-49 | -0.038^***^ | -0.022 | 0.017 |
|  | (0.010) | (0.015) | (0.013) |
| hus. age 50-59 | -0.034^**^ | -0.022 | 0.027^+^ |
|  | (0.011) | (0.019) | (0.015) |
| hus. edu. high school | 0.087^***^ | 0.10^***^ | 0.052^***^ |
|  | (0.0076) | (0.0091) | (0.011) |
| hus. edu. university | 0.11^***^ | 0.17^***^ | 0.079^***^ |
|  | (0.016) | (0.012) | (0.011) |
| hus. minority | -0.034^*^ | -0.040^+^ | -0.014 |
|  | (0.015) | (0.023) | (0.018) |
| Husband's income^2 | -0.000000026^***^ | -4.5e-09^***^ | -5.5e-10^*^ |
|  | (4.2e-09) | (1.0e-09) | (2.2e-10) |
| Husband's income^3 | 7.1e-13^***^ | 4.8e-14^***^ | 5.5e-15^*^ |
|  | (1.8e-13) | (1.2e-14) | (2.4e-15) |
| Observations | 36012 | 16246 | 17422 |
| *R*^2^ | 0.077 | 0.092 | 0.042 |

All regression include wifes' potential income at each 5th percentile and year dummies.

Source: Authors' own based work based on ENEMDU.

Robust standard errors in parenthesis are clustered at demographic group level.

+ p<0.10, * p<0.05, ** p<0.01, *** p<0.001

*Table A 13: Complete Set of Coefficients for the Specifications Reported in Table 5 (c)*

|  | (1) | (2) | (3) |
| --- | --- | --- | --- |
| Dependent variable  Part-time Job | Primary | Secondary | University |
| Prob. wife earns more | 0.020 | 0.076^***^ | 0.063^***^ |
|  | (0.014) | (0.017) | (0.013) |
| age 30-39 | -0.022^*^ | -0.040^***^ | -0.032^**^ |
|  | (0.011) | (0.011) | (0.011) |
| age 40-49 | -0.031^*^ | -0.031^+^ | -0.020 |
|  | (0.012) | (0.017) | (0.016) |
| age 50-59 | -0.031^*^ | -0.016 | -0.0025 |
|  | (0.014) | (0.025) | (0.021) |
| Minority | -0.035^*^ | -0.012 | -0.021 |
|  | (0.017) | (0.026) | (0.021) |
| highland | -0.12^***^ | -0.058^***^ | -0.012^*^ |
|  | (0.0067) | (0.011) | (0.0063) |
| Husband's income | -0.00012^***^ | -0.000020^*^ | 0.000020^***^ |
|  | (0.000019) | (0.0000088) | (0.0000056) |
| hus. age 30-39 | 0.027^*^ | 0.033^*^ | -0.011 |
|  | (0.013) | (0.014) | (0.013) |
| hus. age 40-49 | 0.035^*^ | 0.047^**^ | -0.00059 |
|  | (0.014) | (0.016) | (0.014) |
| hus. age 50-59 | 0.030^+^ | 0.039^*^ | -0.012 |
|  | (0.015) | (0.019) | (0.015) |
| hus. edu. high school | -0.034^***^ | -0.065^***^ | -0.054^***^ |
|  | (0.0076) | (0.0088) | (0.011) |
| hus. edu. university | 0.00038 | -0.062^***^ | -0.056^***^ |
|  | (0.016) | (0.010) | (0.011) |
| hus. minority | -0.031^+^ | 0.0073 | -0.0032 |
|  | (0.017) | (0.024) | (0.020) |
| Husband's income^2 | 0.000000012^**^ | 9.9e-10 | -9.3e-10^***^ |
|  | (4.0e-09) | (7.3e-10) | (2.2e-10) |
| Husband's income^3 | -2.8e-13^+^ | -1.1e-14 | 8.4e-15^***^ |
|  | (1.6e-13) | (9.0e-15) | (2.1e-15) |
| Observations | 36012 | 16246 | 17422 |
| *R*^2^ | 0.037 | 0.044 | 0.025 |

All regression include wifes' potential income at each 5th percentile and year dummies.

Source: Authors' own based work based on ENEMDU.

Robust standard errors in parenthesis are clustered at demographic group level.

+ p<0.10, * p<0.05, ** p<0.01, *** p<0.001

*Table A 14:Complete Set of Coefficients for the Specifications Reported in Table 5 (d)*

|  | (1) | (2) | (3) |
| --- | --- | --- | --- |
| Dependent variable Tenure/Long-term Job | Primary | Secondary | University |
| Prob. wife earns more | -0.12^***^ | -0.10^***^ | -0.12^***^ |
|  | (0.0098) | (0.017) | (0.018) |
| age 30-39 | 0.00048 | 0.019^+^ | 0.041^**^ |
|  | (0.0074) | (0.011) | (0.014) |
| age 40-49 | -0.0055 | 0.016 | 0.067^**^ |
|  | (0.0077) | (0.014) | (0.021) |
| age 50-59 | -0.0018 | 0.022 | 0.064^*^ |
|  | (0.0091) | (0.018) | (0.028) |
| Minority | -0.0049 | 0.00094 | -0.00041 |
|  | (0.012) | (0.022) | (0.028) |
| highland | 0.0067^+^ | 0.015^+^ | 0.0036 |
|  | (0.0037) | (0.0079) | (0.0093) |
| Husband's income | -0.000051^***^ | -0.000011 | -0.000026^**^ |
|  | (0.000012) | (0.000011) | (0.0000080) |
| hus. age 30-39 | -0.031^***^ | -0.023^+^ | 0.027^+^ |
|  | (0.0090) | (0.013) | (0.015) |
| hus. age 40-49 | -0.047^***^ | -0.062^***^ | 0.032 |
|  | (0.0096) | (0.016) | (0.020) |
| hus. age 50-59 | -0.064^***^ | -0.062^***^ | 0.037^+^ |
|  | (0.010) | (0.018) | (0.022) |
| hus. edu. high school | 0.038^***^ | 0.034^***^ | 0.065^***^ |
|  | (0.0052) | (0.0070) | (0.012) |
| hus. edu. university | 0.046^***^ | 0.064^***^ | 0.063^***^ |
|  | (0.011) | (0.0096) | (0.013) |
| hus. minority | -0.019^+^ | -0.011 | 0.00046 |
|  | (0.011) | (0.021) | (0.028) |
| Husband's income^2 | 8.9e-09^**^ | 7.5e-10 | 9.5e-10^*^ |
|  | (2.9e-09) | (1.0e-09) | (4.1e-10) |
| Husband's income^3 | -3.3e-13^**^ | -7.7e-15 | -9.7e-15^*^ |
|  | (1.2e-13) | (1.3e-14) | (4.4e-15) |
| Observations | 36012 | 16246 | 17422 |
| *R*^2^ | 0.033 | 0.036 | 0.074 |

All regression include wifes' potential income at each 5th percentile and year dummies.

Source: Authors' own based work based on ENEMDU.

Robust standard errors in parenthesis are clustered at demographic group level.

+ p<0.10, * p<0.05, ** p<0.01, *** p<0.001
